# A cross-sectional study investigating the relationship between urinary albumin creatinine ratio and abdominal aortic calcification in adults

**DOI:** 10.3389/fcvm.2024.1352921

**Published:** 2024-03-04

**Authors:** Xian Xue, Chen Li, Dongping Chen

**Affiliations:** ^1^ Nanyang City Center Hospital, Nanyang, China; ^2^ Nanyang Second General Hospital, Nanyang, China; ^3^ Shuguang Hospital, Shanghai University of Traditional Chinese Medicine, Shanghai, China

**Keywords:** urinary albumin creatinine ratio, abdominal aortic calcification, cross-sectional study, National Health and Nutrition Examination Survey (NHANES), atherosclerosis

## Abstract

**Introduction:**

The presence of abdominal aortic calcification (AAC) is strongly linked to the development of atherosclerosis and the incidence of morbidity and mortality related to cardiovascular diseases (CVD). Urinary albumin creatinine ratio (UACR) was found related with the increased risk of CVD. The aim of this study is to explore the relationship between the UACR and severe AAC (SAAC).

**Methods and Results:**

This study included a total of 2,379 individuals aged over 40 years, and their information was obtained from the National Health and Nutrition Examination Survey conducted (NHANES) in 2013–2014. The measurement of AAC was conducted through dual-energy x-ray absorptiometry and assessed using the Kauppila scoring system. SAAC was characterized by a Kauppila score of 6 or higher. Multivariate regression models were used to analyze the relationship between UACR level and SAAC, with covariate adjustment. In the completely adapted model, the top third subgroup exhibits increased likelihood of SAAC (odds ratio 1.50; 95%CI: 0.98, 2.29; *p* = 0.030) in contrast to the bottom third subgroup. The subgroup analyses revealed a more pronounced correlation among the older participants (*p*-value for interaction = 0.013).

**Discussion:**

In the United States, SAAC was more likely to occur in adults who had a higher probability of UACR. The use of UACR has the potential to be a valuable method for forecasting the likelihood of SAAC.

## 1 Introduction

Cardiovascular disease is a highly fatal illness globally, and atherosclerosis is recognized as a major contributor to cardiovascular diseases (CVD) (1). Abdominal aortic calcification (AAC) serves as an indicator of atherosclerosis and can forecast future morbidity and mortality related to blood vessel health (2–4). A prior investigation discovered that AAC was linked to CVD fatality, and AAC proved to be a more potent prognosticator for overall mortality compared to coronary artery calcium (5). In their study, Namuun Ganbaatar and colleagues found that there was a correlation between the progression of coronary artery calcium and the presence of albuminuria, which was determined by an albumin-to-creatinine ratio exceeding 30 mg/g (6).

Patients may find 24-hour urinary protein collection troublesome and unreliable due to issues with insufficient or excessive collection and laboratory treatment methods, even though it is considered the gold standard for urinary albumin excretion (7). In clinical practice, the urinary albumin creatinine ratio (UACR) is frequently employed as a substitute. Multiple studies have found that UACR is associated with CVD (8, 9), chronic kidney disease (CKD) (10), hypertension (11) and other diseases. There is a wealth of evidence indicating that microalbuminuria (UACR, 30–300 mg/g) and macroalbuminuria (UACR, >300 mg/g) are linked to the advancement of end-stage renal disease (10). Additionally, a study tracking Korean men for 5 years revealed that having high normal albuminuria (UACR <30 mg/g) was a predictor of an elevated likelihood of developing diabetes (12). In the general population, there is a significant association between proteinuria and dyslipidemia (13). Additionally, a direct link has been observed between UACR within the normal range and overall mortality in the general population (8, 14).

However, no studies have been reported on the relationship between UACR and AAC. Our objective was to examine if there is a correlation between normal UACR levels and AAC in the US population during 2013–2014, considering that most individuals in the National Health and Nutrition Examination Survey (NHANES) had UACR within the normal range.

## 2 Methods

### 2.1 Study population and design

**Figure 1:**
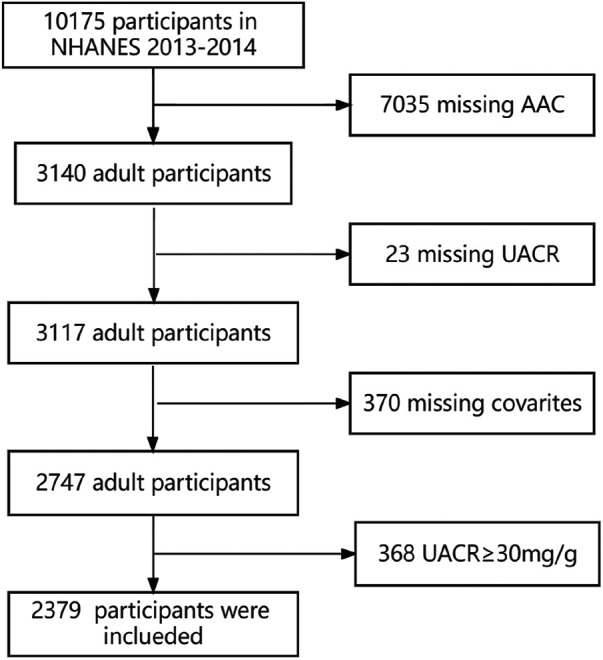
Participant flowchart. NHANES: National Health and Nutrition Examination Survey. AAC: abdominal aortic calcification. UACR: urine albumin creatinine ratio.

The study utilized data from NHANES 2013–2014, a survey that employs sampling design to investigate the health and nutrition of individuals in the United States. NHANES covers five key areas, namely demographic information, physical measurements, examinations, laboratory tests, and questionnaires. In the NHANES study conducted in 2013–2014, a grand total of 10,175 individuals took part. Among them, 7,035 individuals did not have AAC data, 23 individuals lacked UACR data, and 370 individuals lacked covariate data. Additionally, 368 individuals with UACR ≥ 30 mg/g were excluded, leaving a final count of 2,379 individuals included in the study. The data inclusion process is shown in Figure 1. All of them signed informed consent forms.

### 2.2 AAC measurement

As previously reported (15–17), AAC was accurately assessed through a lateral scan of the lumbar spine (vertebrae L1-L4) using dual-energy x-ray absorptiometry (DXA). To measure the degree of AAC, the Kauppila scoring system was utilized (15–17). The AAC-24 scale, which is the accepted technique for evaluating AAC on lateral plain films of the lumbar spine, utilizes a 24-point system (18). This scoring method divides the anterior and posterior aortic walls into four segments, aligning with the regions in front of the lumbar vertebrae L1-L4. Within these 8 segments, aortic calcification is identified visually as either a dispersed white stippling that extends to the anterior and/or posterior aortic walls or as white linear calcification on the anterior and/or posterior walls. The scoring for aortic calcification was as follows: a score of “0” indicated the absence of calcification, a score of “1” indicated calcification of one-third or less of the aortic wall in that segment, a score of “2” indicated calcification of more than one-third but less than two-thirds, and a score of “3” indicated calcification of more than two-thirds. Anterior and posterior aortic walls were scored separately, resulting in a range of “0”–“6” for each vertebral level and “0”–“24” for the total. In view of the previous research (16, 17), severe AAC (SAAC) was characterized as more than 5 score.

### 2.3 UACR

Samples of urine were prepared, kept in suitable frozen (−30°C) conditions, and sent to the University of Minnesota in Minneapolis, MN for examination.

### 2.4 Covariates

The covariates consisted of the following variables: gender (male or female), age, race (Mexican American, Other Hispanic, Non-Hispanic White, Non-Hispanic Black or Other Race), body mass index (BMI), waist circumference, smoking status (participants who had smoked at least 100 cigarettes in their lifetime were considered smokers), alcohol consumption (participants who had consumed at least 12 alcoholic drinks per year were considered drinkers), levels of alanine aminotransferase (ALT), aspartate aminotransferase (AST), serum creatinine (Scr), serum uric acid, total calcium,phosphorus,25OHD_3_, albumin, alkaline phosphatase(ALP), hemoglobin (Hb), hemoglobin A1c (HbA1c), total cholesterol (TC), and diabetes status (participants who were told by a doctor that they have diabetes), hypertension status (participants who were informed by a doctor or other health professional that they have high blood pressure), and systemic immune-inflammationindex [SII, which is calculated as P multiplied by N divided by L, where P represents platelet count, N represents neutrophil count, and L represents lymphocyte count (10^9^/L)]. The SII is closely associated with cardiovascular death and all-cause death according to previous studies (19, 20). The estimated glomerular filtration rate (eGFR) was calculated using the equation from the Modification of Diet in Renal Disease (MDRD) study (21).

### 2.5 Statistical analysis

The analysis was conducted using R Statistical Software (Version 4.2.2, http://www.R-project.org, The R Foundation) and the Free Statistics analysis platform (Version 1.8, Beijing, China). A *P*-value less than 0.05 was considered statistically significant. The UACR was categorized into three groups, with the first group serving as the reference. The initial explanations, categorized by AAC stratification (without SAAC and SAAC), were presented as a ratio for categorical factors, average ± standard deviation or median for continuous factors. Multivariate logistic regression models were used to explore the independent relationship between UACR and ACC in three different models. The adjusted regression model Ⅰ incorporated variables such as age, gender, and race. The adjusted regression model Ⅱ incorporated variables such as age, sex, race, diabetes, high blood pressure, alcohol consumption, smoking, body mass index (BMI), and waist circumference. In the adjusted regression model Ⅲ, besides the above variable, HbA1c, Hb, albumin, AST, ALT, ALP, total cholesterol, uric acid, and serum creatinine, total calcium, phosphorus, 25OHD_3_, SII and eGFR were analysed. Stratified Logistic regression models were utilized to perform the subgroup analyses. The likelihood ratio test was employed to examine interaction among subgroups.

## 3 Results

The baseline characteristics were shown in Table 1. Total 2,379 participants were separated into SAAC group (225, 9.5%) and without SAAC group (2,154). The average age was 58 years. Participants with SAAC appeared to be older, Caucasian compared to those without SAAC. Also there was a significant difference in BMI, 25OHD_3_, serum total folate, total calcium, serum uric acid, Scr, TC, HbA1c, ALT, Hb, SII, eGFR among the two groups. The participants in SAAC group had the lowest levels of TC, Hb, BMI, ALT, eGFR; had the highest levels of 25OHD_3_, folate, Scr, serum uric acid, HbA1c, UACR; had the higher prevalence of hypertension, DM and smoking.

**Table 1:** The participants’ baseline characteristics from NHANES 2013-2014 categorized by AAC group.

Variables	Total (*n* = 2,379)	Without SAAC (*n* = 2,154)	SAAC (*n* = 225)	*p*
25OHD_3_ (nmol/L)	66.4 ± 29.1	65.6 ± 28.4	73.3 ± 34.4	<0.001
Serum total folate (nmol/L)	41.8 (28.5, 61.7)	41.4 (28.2, 60.0)	46.1 (32.1, 79.7)	<0.001
Total calcium (mg/dl)	9.5 ± 0.4	9.5 ± 0.4	9.5 ± 0.4	0.014
Scr (mg/dl)	0.9 (0.7, 1.0)	0.9 (0.7, 1.0)	0.9 (0.8, 1.1)	<0.001
Serum phosphorus (mg/dl)	3.8 ± 0.6	3.8 ± 0.6	3.8 ± 0.6	0.234
Serum uric acid (mg/dl)	5.4 ± 1.3	5.4 ± 1.3	5.6 ± 1.4	0.031
TC (mg/dl)	195.5 ± 40.9	196.3 ± 41.0	187.4 ± 39.2	0.002
HbA1c (%)	5.6 (5.3, 6.0)	5.6 (5.3, 6.0)	5.8 (5.4, 6.2)	<0.001
Gender (*n*, %)				0.768
Male	1,143 (48.0)	1,037 (48.1)	106 (47.1)	
Female	1,236 (52.0)	1,117 (51.9)	119 (52.9)	
Age (years)	58.0 (48.0, 67.0)	56.0 (47.0, 65.0)	73.0 (65.0, 80.0)	<0.001
Race (*n*, %)				<0.001
Mexican American	316 (13.3)	296 (13.7)	20 (8.9)	
Other hispanic	241 (10.1)	229 (10.6)	12 (5.3)	
Non-hispanic white	1,095 (46.0)	951 (44.2)	144 (64)	
Non-hispanic black	418 (17.6)	390 (18.1)	28 (12.4)	
Other race	309 (13.0)	288 (13.4)	21 (9.3)	
Hb (g/dl)	14.0 ± 1.5	14.0 ± 1.5	13.8 ± 1.4	0.013
BMI (kg/m^2^)	28.4 ± 5.5	28.6 ± 5.6	26.8 ± 4.0	<0.001
UACR (mg/g)	9.0 ± 6.0	8.7 ± 5.8	11.7 ± 6.9	<0.001
Waist (cm)	99.1 ± 13.5	99.2 ± 13.7	97.7 ± 10.4	0.093
Albumin (g/dl)	4.2 ± 0.3	4.2 ± 0.3	4.2 ± 0.3	0.463
ALP (IU/l)	64.0 (52.0, 77.0)	64.0 (52.0, 77.0)	62.0 (51.0, 75.0)	0.390
AST (IU/l)	23.0 (20.0, 27.0)	23.0 (20.0, 27.0)	23.0 (19.0, 27.0)	0.991
ALT (IU/l)	21.0 (16.5, 27.0)	21.0 (17.0, 28.0)	19.0 (15.0, 24.0)	<0.001
Hypertension (*n*, %)	1,055 (44.3)	901 (41.8)	154 (68.4)	<0.001
Smoker (*n*, %)	1,079 (45.4)	945 (43.9)	134 (59.6)	<0.001
Drinker (*n*, %)	1,711 (71.9)	1,544 (71.7)	167 (74.2)	0.420
Diabetes Mellitus (*n*, %)	316 (13.3)	267 (12.4)	49 (21.8)	<0.001
SII (1,000 cells/ul)	439.2 (312.7, 608.0)	435.5 (309.3, 597.2)	480.0 (333.9, 678.9)	0.003
eGFR (ml/min/1.73 m^2^)	79.6 ± 19.8	80.7 ± 19.4	69.2 ± 20.3	<0.001

NHANES, National Health and Nutrition Examination Survey; AAC, abdominal aortic calcification; SAAC, severe abdominal aortic calcification; Scr, serum creatinine; TC, serum creatinine; HbA1c, hemoglobin A1c; Hb, hemoglobin; BMI, body mass index; UACR, urinary albumin creatinine ratio; ALP, alkaline phosphatase; AST, aspartate aminotransferase; ALT, alanine aminotransferase; SII, systemic immune-inflammationindex; eGFR, estimated glomerular filtration rate.

Data are shown as mean SD, median (IQR), or *n* (%).

Table 2 shows the logistic regression analysis between different UACR levels and the SAAC. From the Tertile 1 to Tertile 3 level, the prevalence of the SAAC was gradually rised. The risk of SAAC increased as the UACR rose in both the crude model and the adjusted models I and III. In Model III, the odds ratios (ORs) for participants in the second and third tertiles of UACR were 0.99 [95% confidence interval (CI) 0.63, 1.56] and 1.5 (95% CI: 0.98, 2.29), respectively, when compared to those in the first tertile. The *p*-value for trend was 0.03.

**Table 2:** Multinomial logistic regression models of SAAC and UACR.

Albumin creatinine ratio tertiles (mg/g)	Number of participant	SAAC (*n*, %)	Crude model OR(95%CI)	Model I OR(95%CI)	Model II OR(95%CI)	Model III OR(95%CI)
	2,379	225 (9.5)				
Tertile 1 (0.26–5.51)	792	44 (5.6)	1 (Ref)	1 (Ref)	1 (Ref)	1 (Ref)
Tertile 2 (5.52–9.42)	791	60 (7.6)	1.4 (0.93,2.09)	1 (0.65,1.54)	0.96 (0.62,1.5)	0.99 (0.63,1.56)
Tertile 3 (9.5–29.78)	796	121 (15.2)	3.05 (2.13,4.37)	1.65 (1.11,2.46)	1.47 (0.98,2.21)	1.5 (0.98,2.29)
*p* for trend			0.030	0.004	0.029	0.030

SAAC, severe abdominal aortic calcification; UACR, urinary albumin creatinine ratio; OR, odds ratio; CI, confidence interval.

Model I was adjusted for age, gender, and race;model II was further adjusted for diabetes, high blood pressure, alcohol consumption, smoking, body mass index and waist circumference; model III was further adjusted for hemoglobin A1c, hemoglobin, albumin,aspartate aminotransferase, alanine aminotransferase, alkaline phosphatase, total cholesterol, uric acid, and serum creatinine, total calcium, phosphorus, 25OHD3, systemic immune-inflammationindex and estimated glomerular filtration rate.

**Figure 2:**
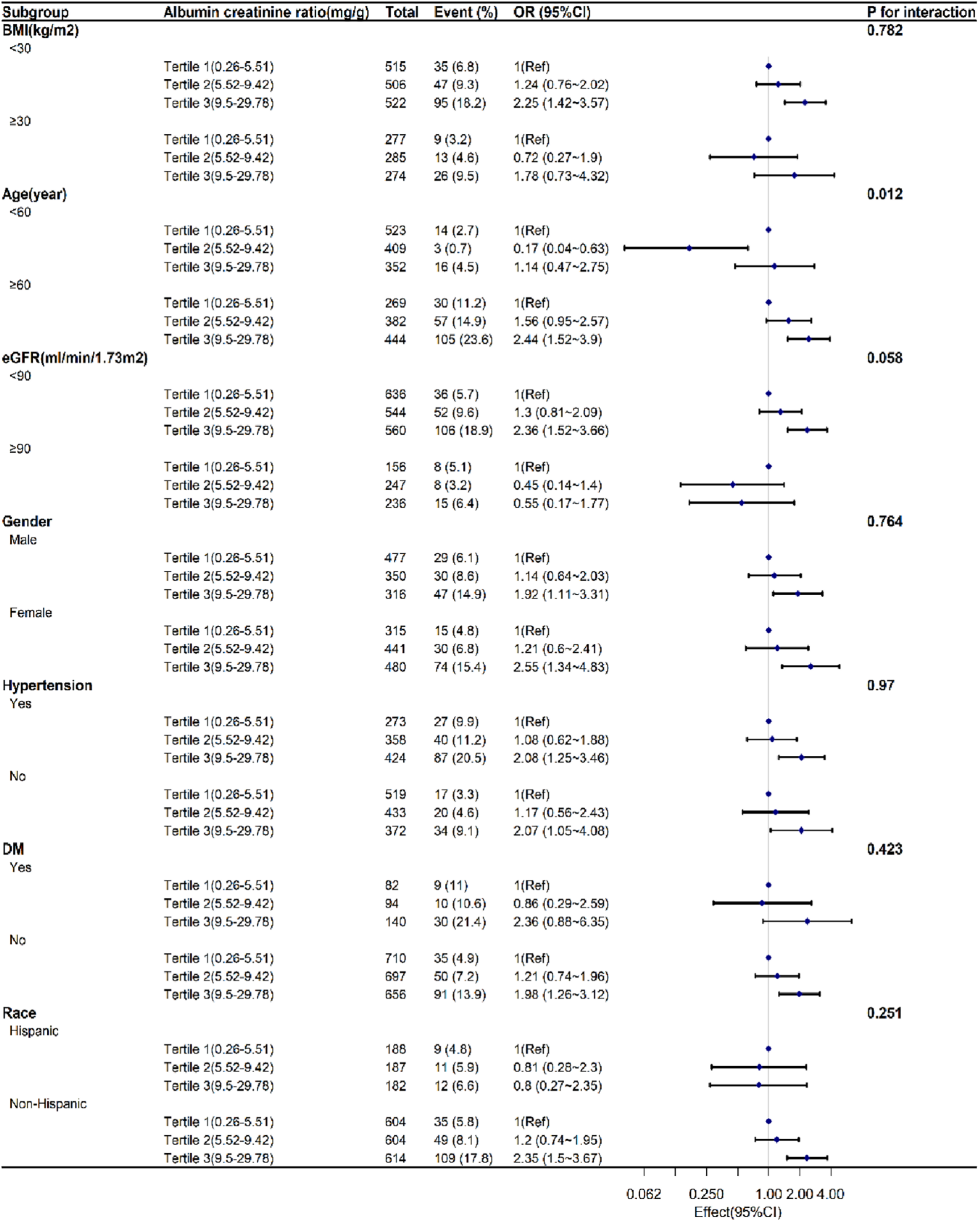
Subgroup analyses exploring the correlation between UACR levels and SAAC.accounting for factors such as age, sex, race, diabetes, high blood pressure, alcohol consumption, tobacco use, body mass index, waist circumference, glycated hemoglobin, hemoglobin, albumin, aspartate aminotransferase, alanine aminotransferase, alkaline phosphatase, overall cholesterol, uric acid, and blood creatinine levels, as well as total calcium, phosphorus, 25-hydroxyvitamin D3, systemic immune-inflammation index, and estimated glomerular filtration rate.UACR:urinary albumin creatinine ratio. SAAC:severe abdominal aortic calcification. OR:Odds Ratio. CI:confidence interval. BMI:body mass index. eGFR:estimated glomerular filtration rate. DM: diabetes mellitus.

Figure 2 displayed the subgroup analyses conducted to assess the stability of the correlation between UACR and SAAC. Only in the age group, we observed the significant difference between the <60 years and the older age (*P *= 0.013). The association was consistent in the gender, BMI, eGFR, Race, hypertension, DM. There was a nearly statistically significant distinction observed between the eGFR<90 mL/min/1.73 m^2^ and ≥90 mL/min/1.73 m^2^ (*P *= 0.065).

## 4 Discussion

The objective of our research was to uncover the correlation between the typical UACR range and the SAAC. Our investigation revealed a direct association between the UACR level and the likelihood of experiencing SAAC.

According to the current guidelines, the UACR concentration above 30 mg/g was considered to be clinically meaningful albuminuria (22). However, the normal range albuminuria was found associated with an increased CVD risk (23), hypertension (24–26), CKD (27) and atherosclerosis (28). In a nondiabetic population with normal-range UACR and eGFR, Aiko Okubo and colleagues discovered that high-normal albuminuria and hypertension were linked to the development of CKD (27). A comprehensive study of a significant population indicated that mild albuminuria (less than 30 mg/g) is linked to mortality from all causes and cardiovascular diseases (29). According to research conducted in Hong Kong, it was discovered that even when the UACR reached levels as low as 1–1.4 mg/mmol (8.4–11.76 mg/g), there was a notable rise in the likelihood of CVD or mortality in both males and females (30). In a cross-sectional investigation conducted in Korea, it was found that patients with type 2 diabetes who had high normal albuminuria showed a significant correlation with atherosclerotic vascular alterations (31). According to a 2005 publication, the UACR has a strong and autonomous correlation with both the existence and seriousness of atherosclerosis in the overall populace, including individuals with normal levels (28).

While the UACR was acknowledged as a possible contributor to cardiovascular disease, the precise mechanisms behind it are still not fully understood. A possible mechanism is that the UACR is associated with permeability of multiple capillary beds and endothelial dysfunction (32). Other study found that low-grade inflammation is related with the UACR (33, 34). The development of increased UACR, endothelial dysfunction, and chronic inflammation occur simultaneously and progress over time (35). Vascular calcification can be facilitated by chronic inflammation (36, 37).

In older participants, the subgroup analysis revealed a stronger correlation between UACR and SAAC. The process of getting older may cause dysregulation of the immune system, which can lead to chronic inflammation at a low level (38). This, in turn, increases the vulnerability to chronic illness, disability, frailty, and early mortality (39). Besides, a stronger connection was found in the eGFR below the 90 ml/min/1.73 m^2^. Individuals suffering from CKD and end-stage renal disease experience notable cardiovascular morbidity and mortality, which can be attributed, at least in part, to the occurrence of vascular calcification (40). These reminds us that the UACR should be concerned in older and CKD patients.

There are certain constraints associated with this research. To clarify the causality, it is necessary to conduct prospective research with larger sample sizes as the cross-sectional study design did not allow us to determine a causal relationship. Furthermore, the individuals involved in our study were recruited exclusively from one nation, and their ethnic background may not be applicable to numerous countries across the globe. Third, only individuals aged 40 and above were included in the acquisition of AAC data obtained from NHANES.

## 5 Conclusion

According to our research, the UACR within the normal range was linked to the likelihood of experiencing SAAC. Identifying patients at risk of AAC is crucial, as emphasized by the current results, which underscore the significance of managing the UACR within the normal range. Nevertheless, additional investigation is still necessary to validate our discoveries.

## Data Availability

The raw data supporting the conclusions of this article will be made available by the authors, without undue reservation.
